# Using ontologies to describe mouse phenotypes

**DOI:** 10.1186/gb-2004-6-1-r8

**Published:** 2004-12-20

**Authors:** Georgios V Gkoutos, Eain CJ Green, Ann-Marie Mallon, John M Hancock, Duncan Davidson

**Affiliations:** 1 Bioinformatics Group, MRC Mammalian Genetics Unit, Harwell, Oxfordshire, OX11 0RD, UK; 2 MRC Human Genetics Unit, Edinburgh, EH4 2XU, UK

## Abstract

The mouse is an important model of human genetic disease. Describing phenotypes of mutant mice in a standard, structured manner that will facilitate data mining is a major challenge for bioinformatics. Here we describe a novel, compositional approach to this problem which combines core ontologies from a variety of sources. This produces a framework with greater flexibility, power and economy than previous approaches. We discuss some of the issues this approach raises.

## Abstract 2

By combining ontologies from different sources the authors developed a novel approach to describing phenotypes of mutant mice in a standard, structured manner.

## Background

Mutant mice are the premier genetic models for human diseases. An increasing number of laboratories and companies worldwide are now carrying out detailed analyses of mouse phenotypes that have been generated from large-scale mutagenesis of the mouse genome. Description of mouse phenotypes has not traditionally adhered to predefined rules or been recorded in databases. However, the sheer volume of data from high-throughput screens (such as *N*-ethyl-*N*-nitrosourea (ENU) mutagenesis [1]) is now driving the need to manage information about mutants in a paperless environment and to build databases that will allow this data to be shared between laboratories and used to formulate hypotheses about gene function. The key to satisfying this need is the ability to describe different phenotypes in a consistent and structured way. There is a need for consistency in the way different communities of biologists attempt to present this kind of data since consistent representation of phenotypes across different domains (such as pathology and anatomy) and species is crucial for the semantic interpretation and the efficient use of this complex information in different kinds of study, such as comparison of gene functions between species.

Ontologies have been an important tool for structuring biological information since the time of Linnaeus. With the advent of the Gene Ontology (GO) in 2000 [2] these techniques for strictly specifying the semantic relationships between terms have become a standard to support knowledge representation in the field of genomics. Hierarchical ontologies hold information about the structure of a particular domain of knowledge at varying degrees of detail (granularity), thus permitting us to integrate concepts and descriptions at different levels of resolution. This approach is forming the basis of new methods for mining biological data [3,4]. In this article, we describe developments in describing mouse phenotypes using ontologies.

### Ontologies and knowledge bases

The term ontology is derived from the Greek and is used in philosophy to mean 'a description of what exists'. There are many definitions of the word, however, and for the purpose of this article, an ontology is 'a specification of entities and their relationships' [5]. The key word 'specification' implies a formal organization. Thus, an ontology is a formalism to describe entities and the relationships between them. Ontologies for computing applications are schemas for metadata [6]. They provide a controlled organization of terms and their relationships that has explicitly defined and machine-processable semantics [7]. The controlled semantic portrayal of entities and their relationships allows the description of a domain of knowledge. For our purposes ontologies mainly attempt to replace free-text descriptions of phenotypes with equivalent computable descriptions that can be used to draw inferences about these data.

An ontology together with a set of individual instances of the kinds of entities it specifies constitutes a knowledge base [8]. It may be difficult to distinguish between the knowledge contained in an ontology and the knowledge contained in a knowledge base [9]. In phenotype ontologies the distinction between the ontology and the knowledge base must be clear. The ontology should capture the general conceptual structures necessary to describe the domain, whereas the knowledge base should provide the individual instances that are described using the ontology. So, in the ontology one can first define the entity (class) of 'pain perception' and further, assign to this entity the attribute 'relative sensitivity' and specify for this attribute a range of allowed values using concepts such as 'sensitive' or 'insensitive', and so on, thereby allowing us to describe pain-perception phenotypes. The knowledge base, however, holds data about particular instances [10], for example a particular mouse with a particular genotype, under defined handling conditions and of a certain age, that has a particular level of sensitivity to pain according to a particular assay. In other words, the ontology constitutes a general theory (how to describe phenotypes), whereas the knowledge base describes particular circumstances, in our case particular instances of phenotype.

### Why use ontologies?

An important question here is why do we need to use ontologies; why not simply use a series of unconnected, standard terms such as provided by a controlled vocabulary? The advantages of using ontologies have been argued extensively, but the main reason is that ontologies are attempting to capture the precise meaning of terms. Furthermore, ontologies can be used for reasoning and inference (for example, consistency checking or drawing conclusions from the knowledge). The most important factor from our perspective is the need to combine information from different phenotypes or from different protocols (assays). For example, if a mutant mouse has six digits in each forelimb we will wish to use this information in a variety of ways (for example, to group mice with limb pattern defects, or with affected forelimbs, or with abnormal numbers of digits in any limb). For this, we need not just a controlled vocabulary of terms, but also information about how these terms relate to one another (for example, that forelimb is an instance of 'limb', that the normal number of digits in the forelimb is five, that the number of digits is an instance of 'pattern', and so on).

### Current approaches to the description of mouse phenotypes

Traditionally, the main source of information for most scientists is the peer-reviewed journal literature. Electronic versions of published information have opened the road to accessing and retrieving information in a much easier and more cost-effective manner. The growth and wider availability of the world-wide web has led to a significant growth in the amount of readily available electronically stored information [11]. With this surge of readily available information the location and retrieval of relevant information has become a major (commercial) activity [12]. One of the most important issues in information retrieval is constructing effective indexing methods that are required for the sophisticated querying of the stored data. Free-text searching forms the basis of information retrieval but is extremely limited because of the inherent lack of accuracy and specificity. Complex free-text descriptions, such as are used for phenotypes, are almost impossible to index and retrieve in a useful way directly from the biomedical literature. The potential power of complex searches against information from multiple experiments requires the annotation of free text into structured representations that can be understood and where the power of computational algorithms can maximize the potential of the information to be compared and contrasted.

The most comprehensive attempt to annotate mammalian phenotypic data so far, the Mammalian Phenotype Ontology (MP) [13], is currently under development by the Jackson Laboratory [14,15]. The current structure of the ontology is generated using DAG-Edit [16], the current GO standard, and allows a hierarchical display of terms and their definitions. These terms include a combination of entities and values, for example, id MP:0001509 corresponds to 'abnormal body position', which at a high level provides a sufficient description of phenotypic data.

This approach allows high-level access to the knowledge held in the ontology, but also has certain limitations similar to the GO paradigm. If one attempts to create too much specificity within an ontology of this type it can expand to unmanageable proportions and parentage relationships can be overlooked as their number grows. For example, merely creating new terms by prepending the two qualifiers, 'increased' and 'decreased', everywhere that is applicable, will massively increase the size of the ontology. To allow a systematic approach to the model, combinations would also have to be instantiated that might never be used. Because there is a practical limit to the number of values that can be managed, such an approach is limited. Inevitably, decisions have to be made as to which individual combination describes a particular phenotypic entity best.

We note here though that the development of MP is being developed pragmatically, with instances being added as needed to annotate mouse phenotypes, following the paradigm used by GO developers. MP is a cross-product ontology that includes mouse anatomy ontology, GO and other controlled terms as part of the construction of MP terms. Although the cross-reference IDs are not visible, they are part of the design of MP. Some of the work described here reflects insights gained during extensive discussions about the representation of phenotypes at the Phenotype Consortium meeting held in Bar Harbor, ME in September 2003. The developers of the MP ontology are part of this consortium and have intentionally created their ontology in such a way that it can be easily extended to form instances of the compositional approach discussed in the next section.

With the objective of capturing information about phenotypes in any organism, Ashburner proposed the Phenotype And Trait Ontology (PATO) [17] in 2002. PATO is a schema according to which, "phenotypic data can be represented as qualifications of descriptive nouns or nounal phrases" (M. Ashburner, unpublished work). Each noun represents an observable characteristic and for each noun there will be a set of attributes, for each of which is defined a set of appropriate values. In addition to these three semantic classes (namely observable entities together with the associated attributes and values), the concepts that are needed to describe phenotypes include the assays by means of which the phenotypes were determined and the environmental and genetic conditions (Microarray Gene Expression Data Society [18]) under which these assays were performed. Taken together, the semantic concepts and relationships defined for PATO, assays, genetic and environmental conditions, will form the basis for the systematic description of phenotypes.

## Results

### A proposal for describing mouse phenotypes

**Figure 1:**
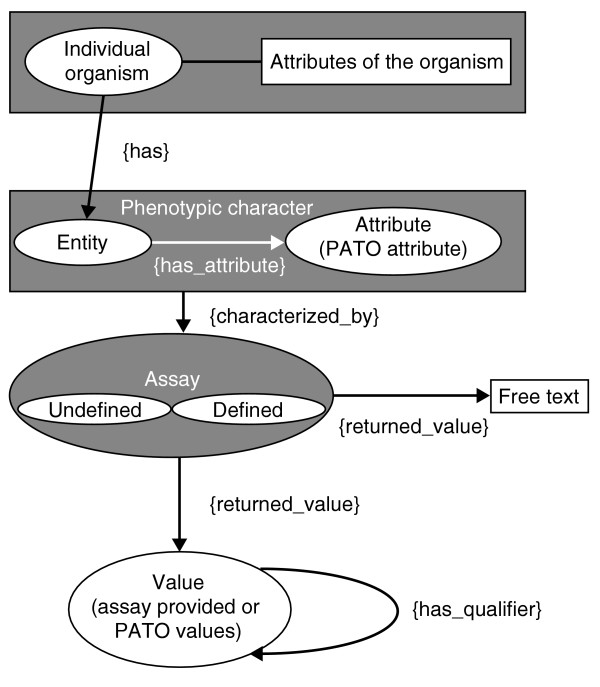
Proposed schema for constructing phenotype ontologies (modified from [13]).

The description of mutant phenotypes must provide a practical way to capture the biologically relevant information about the phenotype in machine-readable form [19]. It should allow us to compare, combine and analyze different phenotypes. For this, the ontology must first be consistent, and second be able to generate statements that have a logically well-formed structure in order to support reasoning from descriptions of different phenotypes. To provide these functionalities we propose a compositional method of describing phenotypes [19]. By this we mean that the description of the phenotype combines terms from different standard ontologies, each of which supports a particular domain of knowledge. A list of ontologies that should be included in such a phenotype ontology is given in Table 1. These ontologies are combined in a specified formula or schema that provides the logical structure of the whole. The schema itself can be considered as a meta-ontology that describes how other ontologies relate to one another. Figure 1 illustrates such a schema.

**Figure 2:**
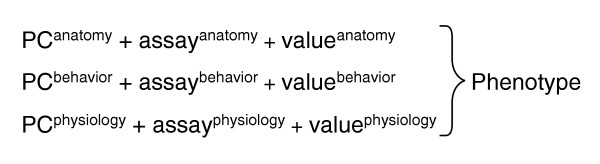
Schematic of phenotype description as the sum of the results of assaying different characters. PC, phenotypic character.

According to the schema in Figure 1, the whole organism has certain attributes, such as genotype, identity number, and exists under certain handling conditions (Table 2). The organism also has a set of core components including its anatomy, development, physiology and behavior. Each of these core components is represented by a separate ontology and each has a set of attributes, again represented by an ontology. For example, the organism may have an anatomical component 'left eye' which is a term from the anatomy ontology. The left eye, in turn, may have attributes of 'color', 'size', and so on, taken from the attributes ontology. This combination of core entity and attribute constitutes a phenotypic character - something that can be measured. Phenotypic characters, in turn, link to 'assays', which return a variety of 'values', again represented by an ontology, which may be applied to the phenotypic character in question. When this schema is used to describe actual phenotypes, instances of single phenotypic characters are linked together to provide a full phenotypic description of an individual organism. Each character can be represented by a line in a table where the table represents the full phenotype. Figure 2 presents this schematically.

According to the schema in Figure 1, five classes of ontology (in circles), namely organism, entity, attribute, assay and value, are required to express a phenotypic instance.

#### Organism

This class holds the information (organism attributes) of an organism in which the phenotypic characters are observed (see Table 2).

#### Entity

Entities will be formed by importing ontologies discussed in Table 1: behavior, anatomy, and so on. Each entity may be associated with a set of attributes, for example, color and size, that may also be shared with other entities.

#### Attribute

Attributes will be provided by PATO [17]. PATO should hold general attributes that can be applied through different phenotypic ontologies. This has the advantage of economy and also enables cross-referencing between domains. New attributes should be assigned to classes only when they cannot be modeled with existing options.

#### Assay

Assays will have a hierarchical structure and will define a range of values that correspond to a particular combination of entity and attribute (that is, phenotypic character). They hold multiple relations to values, qualifiers and free text as well as their own metadata. The slot for free text is included to capture knowledge that cannot be expressed through the ontology as yet.

#### Values

Splitting PATO into two different ontologies, PATO attributes (above) and PATO values, allows the PATO ontology to be incorporated into the schema [19]. Values can thus be either specific values provided by the assay or common values, provided by PATO. A possible relationship between these sets of values would be 'interpretation_of'. Although values provided directly by the assay are usually the objective recordings of a test for a specific phenotypic character, there can be an interpretation of these recordings in terms of a higher level phenotypic character. For example, in an assay of memory in the mouse that uses a water test, the values returned by the test may be that a mouse completed the task in a certain time and manner, but these results may be interpreted to indicate a value corresponding to the phenotypic character comprised by the entity 'memory' that was assayed for the attribute of 'short-term recall' and returned the interpretative value 'loss of memory'. By introducing the 'interpretation_of' relationship, we could make this distinction in a machine-understandable manner and allow the possibility, if required, of expressing the original objective values of the test, thus avoiding information loss. This aspect of the schema remains under study.

A central idea in this schema is that of the 'phenotypic character', which we can define as any feature of the organism that is observed or 'assayed'. An example for the mouse is tail length. A phenotypic character is a compound composed of an entity, in this case an anatomical entity 'tail', and an attribute of tail, here 'length'. Similarly the physiological entity 'hearing' (GO:0007605) has the attributes 'sensitivity', 'range', and so on. Thus, 'hearing range' and 'hearing sensitivity' are distinct phenotypic characters. The ideal phenotypic character is one that can be measured independently of others. In practice, however, phenotypic characters are rarely independent. Furthermore, the observations from any particular assay will most probably depend on several different phenotypic characters. For example, the results returned by the click-box test for hearing sensitivity in the mouse actually depend, not only on hearing, but also on the mouse's ability to make a detectable locomotor response (the Preyer reflex [20]).

These multiple dependencies are captured in the schema, enabling the ontology to support the appropriate possible groupings of phenotypes. This will allow us, for example, to group all mutants that have (by direct assay), or may have (for example, those failing the click-box test), an effect on the locomotor system. Conversely, different assays may provide information about a single phenotypic character. For example, an acoustic brain-stem response (ABR, a sound-evoked potential within the acoustic nerve) [21] can be measured to assay basic hearing ability as well as to give a threshold-response curve for differing frequencies. Linking assays with characters in this way will support machine reasoning, enabling us, for example, to make the hypothesis that a particular mouse has a locomotor rather than hearing defect. Indeed, the need to capture this network of relationships between assays and phenotype is a strong indication of the need for an ontology rather than merely a controlled vocabulary of unrelated terms.

The expressivity of representation languages such as DAML+OIL [22], OWL [23] and OBO [17] could also dynamically account for the possibility of a cross product or dependence required for representing a phenotype. For example, if a cross product between ontologies does not exist (that is, one of the required terms is not to be found in an ontology), one can assign an 'anonymous class' that is dynamically defined as being both a class in one case and an instance in another. As an example, one might want to refer to the term cocaine dependence, but that cross product may not exist. An 'anonymous class' can be dynamically defined as being both 'cocaine' (coming from a chemical ontology) and 'dependence' (coming from the behavior ontology) to generate this cross product.

Finally, we note here that it should be possible to link current high-level structures (such as the current MP ontology), which are necessary in many cases for annotation purposes, to the more expressive form we propose here, so that it can also be explored computationally.

### Example

In this section we describe an example of the application of the compositional schema. We chose a phenotype example at random from the MP database: 'nest building' [MP:0001447]. Several descriptions of nest-building patterns can be found in the corresponding reference [24]. For example, the authors comment: "Note the fluffy well formed nests built in the +/+ cages and the huddling of mice in these nests, in contrast to the poorly formed nests in -/- cages with random sleeping patterns." and later: "In addition, +/+ mice built nests from nestlet material that averaged 50 mm in depth, while -/- mice built significantly shallower nests (Figure 4D), with depths that averaged less than 20 mm [*t*(10) = 3.754, *p *< 0.004]." The authors also describe the assays used to record these observations: "Nesting Patterns: six cages of wild-type and six cages of mutant mice (*N *= 4 mice per cage) were used to evaluate nesting patterns. A 5 × 5 cm piece of cotton nesting material (Ancare, Bellmore, NY) was placed in each cage. After 45 min, photographs were taken of each nest and the nest depth was measured. Nest height data were analyzed using the Student's *t *test."

For some users/applications the compound term 'abnormal nest building' might be a sufficient description of this particular phenotypic instance, but this would result in information loss. A human would have to retrieve and read the reference to extract further information. Our schema allows the expression of this information in a machine and human readable manner. In Table 3 we provide the relevant part of our ontology modeled according to the schema. One can easily express these phenotypic instances. In order to describe fluffy, well formed nests or poorly formed nests one would use the following combination:

*Nest building *{has_attribute} *attribute:quality *{characterized_by} *defined_quality_assay *(described in Nesting Patterns [24]) {returns_value} *well-formed*

*Nest building *{has_attribute} *attribute:quality *{characterized_by} *defined_quality_assay *(described in Nesting Patterns [24]) {returns_value} *poorly-formed*

*Nest building *{has_attribute} *attribute:quality *{characterized_by} *defined_quality_assay *(described in Nesting Patterns [24]) {returns_value} *fluffy*

We note here that had the value 'fluffy' not been included in the standard values for a quality assay, it could be captured in the free-text field provided by the schema. To express a nest of 50 mm depth or significantly shallower:

*Nest building *{has_attribute} *attribute:absolute_depth *{characterized_by} *undefined_ absolute_depth_assay *{returns_value} *50 mm*

*Nest building *{has_attribute} *attribute: relative_depth *{characterized_by} *undefined_ relative_depth_assay *{returns_value} *shallow *{has_qualifier} *significant*

With this information one could go back to a higher level and still be able to express a more general characterization of this phenotype as 'abnormal nest building' but obviously the opposite is not possible.

An important unresolved issue concerning the use of ontologies to describe phenotypes arises from the fact that all the ontological structures developed so far are designed to describe individual mice. Mutagenesis experiments usually characterize a number of mutant mice to take into account variable penetrance of the mutation and other stochastic effects. A strategy will therefore need to be developed to describe the generalized phenotypic properties of a cohort of mice. This may involve the use of more sophisticated relations such as {usually characterized by} or even quantitative relations such as {80% characterized by}.

## Discussion

### Importance of the assay

The assay plays a central role in our schema (Figure 1). Assays are the means of making observations and as they determine what can be observed they are a necessary complement to the attribute ontology. Generally, they are recorded as protocols or even as standard operating procedures (SOPs). However, even a visual observation is a form of assay and this needs to be reported when one expresses a phenotypic instance, for example:

*eye *{has_attribute} *attribute:color *{characterized_by} *visual inspection *{returned_value} *pink*

On a practical level, assays can add specificity and functionality to the relationship between entities, their attributes and the corresponding values. Most important, an assay vocabulary allows the entire schema to be dynamic by including new assays and capturing explicit differences between assays in different laboratories. The assay will also allow standardization and definition of values for a given phenotypic character, for example, how abnormal is defined in relation to body position.

### Implementation

Our schema can be expressed using a variety of modeling tools and knowledge representation (KR) languages [25]. We chose DAG-Edit [16] (version 1.408) and Protégé-2000 [26] (version 1.9) which is Java-based, well supported and incorporates multiple inheritance, relation hierarchies, meta-classes, constraint axioms and F-Logic [27]. Although the complexity of our current models can be described with existing tools, in the future more complex phenotype domains may require migration to a finer-grained conceptualization.

### Populating the Mouse Phenotype Ontology

**Figure 3:**
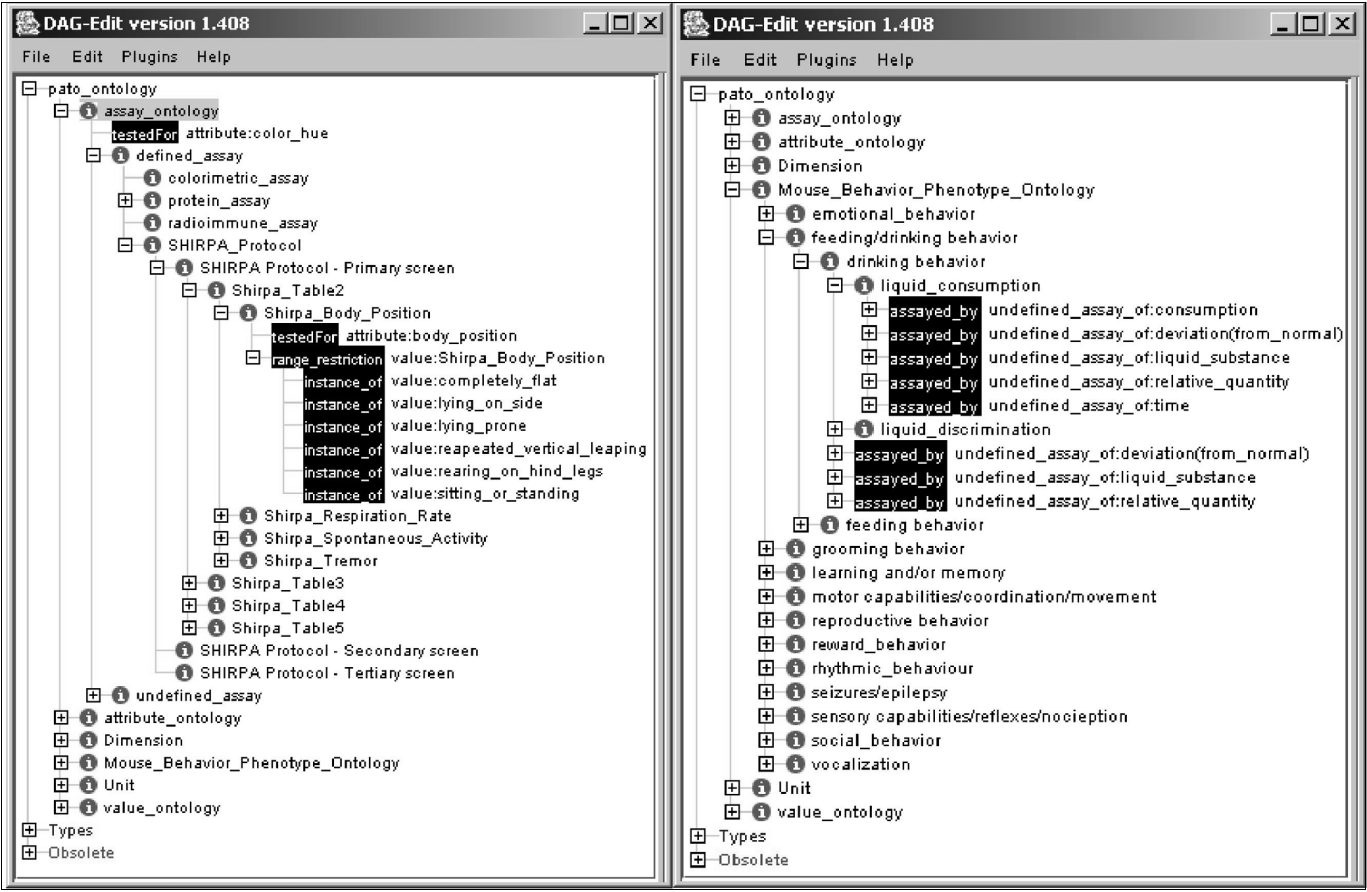
Two snapshots of the ontology visualized using DAG-edit.

**Figure 4:**
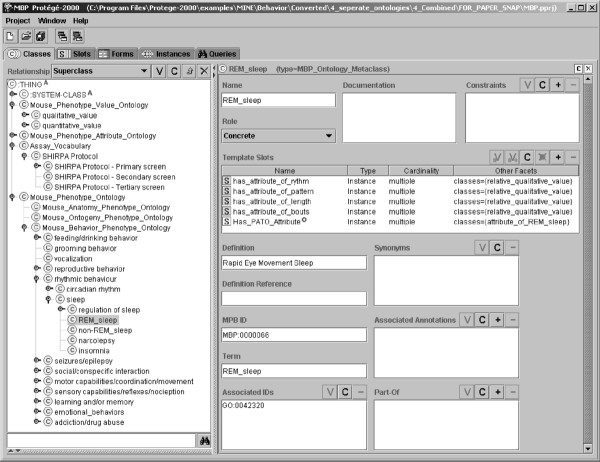
A snapshot of the ontology using Protégé-2000.

The schema was designed to be easily populated using extant core ontologies, such as anatomy, and defining attributes related to each entity. The assay vocabulary can be constructed as required. Permitted values are defined in the range of different assay attributes in part devised in the form of a general scheme and in part built from the output of particular assays. Although we include for demonstration purposes three core ontologies, namely behavior, anatomy, and developmental anatomy (Figures 3 and 4), we have tested the schema only on behavior. We also include a possible structure for PATO attributes and a separate ontology for common values. We note, however, that the structure of PATO has not been finalized. Figure 3 shows the implementation of the schema in DAG-edit.

Figure 4 shows a typical implementation of the Schema in Protégé 2000. Options for providing a definition, definition reference, documentation, associated annotations, synonyms, and so on, are offered in our schema. Similar options can be used for attributes using the metaslot options.

Since most of the ontologies we are planning to use were generated using the DAG-edit [16] format, we had to convert them to the Protégé-2000 format using the tools and methodology described by Yeh *et al*. [27], with minor modifications. This task, however, should no longer be necessary as the latest version of DAG-edit allows the export of ontologies in OWL format.

### Modeling issues

Decisions will inevitably have to be made to combine a core ontology with its attributes and then define facets of that relationship, for example, cardinality, attribute value type and attribute range. In our schema, the class hierarchy of all ontologies employed represents an 'is-a' relation. So, mouse social behavior 'is-a' mouse behavior, or mouse social behavior is a 'kind-of' mouse behavior and so forth. All other relationships, including PATO and 'part-of' relationships, are modeled as attributes. However, we note here that efforts are currently being made by the GO consortium to define and formalize the 'part-of' relationship, which is considered vital and special in bio-ontologies, especially anatomy [28].

Because our phenotype ontology and PATO need to be the result of a collaborative effort within the communities, we feel that it is important to set out the basic modeling concepts that need to be applied upon allocating attributes to the core ontologies. Deciding whether to introduce a new attribute or represent this functionality through an entity is often quite difficult. Several things need to be considered in order to make the best decision, although it should be noted that there are no clear distinction as to what is a right or wrong decision.

The first thing to take into account is that subclasses of a class inherit all properties of the parent and could have additional properties and different restrictions from the latter. PATO should remain as general as possible, and, when possible, care should be taken to avoid making PATO domain specific. For example, in the behavior ontology there is a class named 'reflexes' that contains children such as 'blinking reflex', 'Preyer reflex' and 'righting reflex'. It might be worth considering having one 'attribute of reflex' available in PATO rather than creating a separate attribute 'of' for each individual reflex, such as 'attribute of blinking reflex', 'attribute of Preyer reflex', and so on. Then again, if one wishes to assign different functionalities to these properties, creating separate attributes might be useful. As a rule though, one should consider that PATO needs to be consistent, usable and interoperable if it is to be applied to the general domain of phenotypes. Repetition between core ontologies and PATO should be avoided where possible.

What is also often not clear is whether one should add a new class to represent functionality or assign attributes to already existent classes. For example, think of the entity 'body position'. There are several ways to model this entity in the mouse behavior phenotype ontology. One could declare 'body position' as a child of a class called 'posture'. An 'attribute of body position' could then be assigned to this class with a range of values that might be specific to an assay, for example SHIRPA [29] allows the value 'lying on its left side' among other values to an assay for body position. Alternatively, a more general 'attribute of position' could be assigned to this class. The choice depends on the functionality of the ontology and the range of phenotypes we wish to express. If the entity requires more specific attribute values to represent specific functionalities important to the domain of knowledge, we assign more specific attributes. If this functionality is not important for the domain, we assign specific attribute values [8].

'Body position' could also be split into an entity of 'body' and an attribute of 'position'. Again, a new class 'body position' should be assigned, if one considers the objects with different attributes as different kind of object and this distinction important in the domain. As a general rule, before assigning new classes and attributes one should consider the functionality and their role in the domain, creating more distinctions as the depth of knowledge that is required to be expressed in the ontology increases.

Classes in the hierarchy should not necessarily have to introduce new properties [8]. Although, in many cases these entities could be represented as attributes, it is not necessary for the functionality of the domain. If the expert thinks that this distinction is significant for the class hierarchy and the logical representation of his knowledge of the domain, then these entities should be represented as classes [8]. An important additional consideration is whether creating new terms in an ontology results in terms that cannot be consistently distinguished experimentally ('resolution').

## Conclusions

We have presented here an approach to the use of ontologies in describing mouse phenotypes that could provide a platform for the consistent representation of mouse phenotypic data. We have also described in detail a possible methodology to construct applications of this schema across different domains. We have dealt with modeling issues and provide guidelines to deal with semantic and practical problems.

We maintain that such modeling efforts in any domain should be done in a collaborative fashion in the community. Repetition between different parts of the mouse phenotype ontologies is unavoidable. However, the use of consistent IDs, synonyms and records for associated annotations could allow seamless integration of ontology products. The nature of the schema proposed, as well as its components, is extremely dynamic; therefore coordination of efforts is vital.

The structure allows extensibility and interoperability. Although an ontology should not cover all possible information about a domain, the main idea behind this concept is to allow the phenotype ontology to cope with novel and unpredictable phenotypes and account for new assays, serving scientific autonomy and information validity and integrity. We have built a software system [30] which includes a browser that allows searching and viewing the knowledge captured though the complex relations described here and databases that allow the dynamic update of different parts of the core ontologies, including PATO, without the loss of applied facets.

## Figures and Tables

**Table 1:** Ontologies to be incorporated in a combinatorial phenotype ontology

Ontology	Description	URL
Adult anatomy	The Anatomical Dictionary for the Adult Mouse [17] has been developed by Terry Hayamizu, Mary Mangan, John Corradi and Martin Ringwald, as part of the Gene Expression Database (GXD) [31] Project, Mouse Genome Informatics (MGI), The Jackson, Laboratory, Bar Harbor, ME [14]	[17]
		
Developmental anatomy	The Anatomical Dictionary for Mouse Development has been developed at the Department of Anatomy, University of Edinburgh, Scotland (Jonathan Bard) and the MRC Human Genetics Unit, Edinburgh (Duncan Davidson and Richard Baldock) as part of the Edinburgh Mouse Atlas project (EMAP), in collaboration with the Gene Expression (GXD) project at MGI, The Jackson Laboratory, Bar Harbor, ME [31,32]	[17]
		
Behavior	Parts of behavior have been expressed in a consistent manner [13,17]	[13]
		
Pathology	The Pathbase mouse pathology (Paul Schofield) ontology provides a description of mutant and transgenic mouse pathology phenotypes and incorporates 425 known mouse pathologies hierarchically organized as 'instances of' pathological processes [33]	[17]
		
Gene Ontology	GO describes the roles of gene products and allows genomes to be annotated with a consistent terminology (The Gene Ontology consortium 2002) [2]	[17]
Others		

**Table 2:** Organism attributes

id	Identifier for individual (n)
T	Species (for example, NCBI taxonomy browser [34])
G	Genotype
	I: Strain (for example, StrainID from MGI [14])
	S: Genotypic sex
	A: Alleles at named loci (for example, MGI [14])
E	Handling conditions (see EUMORPHIA [35])
D	Age/stage of development (Theiler [36] and other staging criteria, for example EMAP [37])

**Table 3:** Nesting behavior

Entity	Attribute	Assay	Value
Social behavior	1. Attribute:qualitative	Undefined_qualitative_assay	1. Abnormal
			
Huddling behavior	1. Inherited attribute of class Social Behavior		1.
	2. Attribute:huddling_frequency	Undefined_huddling_frequency_assay	2.
	2a. Attribute:relative_huddling_frequency		2a
	2b. Attribute:absolute_huddling_frequency		2b
			
Nesting behavior	1. Inherited attribute of class Social behavior		1.
			
Nest building	1. Inherited attribute of class Nesting behavior		
	1. Attribute:duration	Undefined_duration_assay	1.
	1a. Attribute:relative_duration		1a. Slow, fast
	1b. Attribute:absolute_duration		1b. 45 min
	2. Attribute:height	Undefined_height_assay	2.
	2a. Attribute:relative_height		2a. short_height, tall
	2b. Attribute:absolute_height		2b. 20 mm
	3. Attribute:weight	Undefined_weight_assay	3.
	3a. Attribute:relative_weight		3a. heavy. light
	3b. Attribute:absolute_weight		3b. 10 g
	4. Attribute:quality	Undefined_quality_assay	4. Good, well-formed, poor, fluffy
	4a. Attribute:shattering		4a.
	4b. Attribute:threshability		4b.
	5. Attribute:depth		5.
	5a. Attribute:relative_depth	Undefined_relative_depth_assay	5a. Shallow
	5b. Attribute:absolute_depth	Undefined_absolute_depth_assay	5b. 50 mm
